# Combined fluticasone propionate and salmeterol reduces RSV infection more effectively than either of them alone in allergen-sensitized mice

**DOI:** 10.1186/1743-422X-3-32

**Published:** 2006-05-23

**Authors:** Rajeswari Singam, Prasanna K Jena, Sumita Behera, Gary R Hellermann, Richard F Lockey, Dennis Ledford, Shyam S Mohapatra

**Affiliations:** 1 Division of Allergy and Immunology, Joy McCann Culverhouse Airway Disease Research Center, University of South Florida College of Medicine and James A. Haley VA Hospital, Tampa, FL, USA

## Abstract

**Background:**

Respiratory syncytial virus (RSV) infection is the major cause of bronchiolitis in infants and is a risk factor for the development of asthma. Allergic asthmatics are more susceptible to RSV infection and viral exacerbation.

**Methods:**

Since the effectiveness of corticosteroids in treating RSV infection has been controversial, we tested fluticasone propionate (FP) and salmeterol (Sal) alone versus FP plus Sal (FPS) on RSV-induced airway inflammation. Mice were sensitized and challenged with ovalbumin (OVA) and infected with RSV. Following infection they were treated with FP, Sal, or FPS intranasally and airway hyperreactivity (AHR), inflammation and RSV titers were examined.

**Results:**

The group treated with FPS showed significantly lower AHR compared to the group treated with FP or Sal alone. The group treated with FP alone showed slightly decreased (non-significant) AHR compared to controls. Treatment with FPS resulted in significant decreases in the percentage of eosinophils and neutrophils in bronchoalveolar lavage fluid and in lung pathology compared to FP or Sal. FP alone decreased eosinophils but not neutrophils or lymphocytes, while Sal alone decreased eosinophils and neutrophils but not lymphocytes. FPS treatment of mice infected with RSV in the absence of allergen sensitization resulted in a 50% decrease of RSV titer in the lung and a reduction in neutrophils compared to FP or Sal.

**Conclusion:**

Together, these results indicate that fluticasone in combination with salmeterol is a more effective treatment for decreasing airway hyperreactivity and inflammation than either of them alone in allergen-sensitized, RSV-infected mice.

## Introduction

Asthma is a chronic lung disease with two distinct features – airway inflammation and airway hyperresponsiveness [1,2]. An association between viral upper-respiratory infections (URIs) and exacerbations of asthma has been reported [3,4]. The most commonly identified viruses in these studies include rhinovirus, coronavirus, influenza virus and respiratory syncytial virus (RSV) [5]. RSV is the predominant cause of URIs in infants below 2 years of age and infection may result in bronchiolitis, which is a risk factor for asthma [6-12]. RSV may constitute the earliest trigger for the development of a T-helper type 2 (Th2)-dominant immune response, which is the hallmark of immunopathology in allergic subjects including asthmatics, and also in rodent models [13]. URIs cause a decrease in peak flow that lags behind upper airway symptoms by 1–2 days, with 46% of subjects in one study reporting a two day lag in peak flow reduction [14].

A combination therapy involving a long-acting β2 agonist and an inhaled corticosteroid (ICS) has emerged as an effective asthma management strategy to control persistent asthma [15]. A combination of salmeterol (Sal) and fluticasone propionate (FP) was found to be superior to either of them alone [16,17]. The combination is also significantly more effective than montelukast plus FP or monotherapy with inhaled budesonide [18]. The increased effectiveness of FPS has been attributed to increased activation and translocation to the nucleus of glucocorticoid receptors [19,20]. However, the effect of these drugs on viral exacerbation in allergic asthmatics has not been studied.

The conclusion of the Cochrane Review of available controlled trials of ICS in children with a history of mild episodic viral wheeze was that high dose ICS was partially effective for the treatment of mild episodic viral wheeze of childhood [21]. Since URIs induce exacerbations, β2-agonists may be of specific value in reducing such exacerbations. In an *in vitro *study of *Pseudomonas aeruginosa *infection, a combination of FP and Sal reduced infection and preserved ciliated cells to a greater degree than either alone suggesting synergy between the two agents [22]. Because 80–85% of asthma exacerbations in children are associated with viral infections, early intervention with a combination therapy should have beneficial effects on viral asthma exacerbations.

Since virus-induced exacerbation is accompanied by airway inflammation, we reasoned that the combination of a steroid and a β-2 agonist might provide protection from severe RSV infection and the ensuing asthma exacerbation. This hypothesis was tested in a mouse model of allergen sensitization and RSV infection using OVA as the allergen [1]. Mice with chronic or acute sensitization to OVA were RSV infected and then treated with FP or Sal or the two together (FPS). Airway hyperreactivity (AHR) and pulmonary inflammation were measured five days after infection. The results show that the combination of FP and Sal provides significant protection in terms of both airway hyperreactivity and pulmonary inflammation compared to either of them alone.

## Methods

### Animals

Female BALB/c mice, 4–6 weeks of age were obtained from Charles River and housed under pathogen-free conditions at the University of South Florida Vivarium. All treatment protocols were approved by the USF Institutional Animal Care and Use Committee (IACUC).

### RSV preparation and infection of mice

The A2 strain of human RSV (American Type Culture Collection, Manassas, VA) was propagated in HEp-2 cells (American Type Culture Collection) grown in Eagle's minimal essential medium (Gibco) with 2% FBS. At maximum cytopathic effect, the cells were harvested in the same medium. The suspension was clarified by centrifugation at 700 × g for 10 min at 4°C and the resulting supernatant was layered onto a glycerol gradient and centrifuged at 14,000 × g for 3 hrs at 4°C. The pellet containing virus was resuspended in pre-cooled (4°C) buffer (0.22 μ-filtered 50 mM HEPES (pH 7.5), 100 mM MgSO_4_, and 150 mM NaCl) and stored in aliquots in liquid nitrogen. Viral titers were determined by standard plaque assay combined with immunostaining for RSV. Mice were infected under light anesthesia by intranasal inoculation of RSV (5 × 10^6 ^PFU).

### OVA sensitization, RSV infection and drug treatment

For experiments in which only a single RSV infection was used (therapeutic regimen), mice were sensitized by intraperitoneal injection (i.p.) of OVA on day 1 and by intranasal (i.n.) administration of OVA on days 7 and 9. On day 9 the mice were infected i.n. with of RSV. From day 10 to day 13 they were treated daily i.n. with fluticasone (FP) propionate, salmeterol (Sal), or the two in combination (FPS) at 10 μg per mouse (FP and Sal were obtained from GlaxoSmithKline). Airway hyperreactivity (AHR) was measured on day 14 and on day 15 the animals were sacrificed. For the prophylactic experiments, mice were sensitized by intraperitoneal injection (i.p.) of OVA on day 1 and by intranasal (i.n.) administration of OVA on days 9, 12 and 14. On day 19 the mice were infected i.n. with of RSV. From day 21 to day 27 mice were treated daily i.n. with FP, Sal, or the two in combination (FPS) at 10 μg per mouse. On day 28, mice were reinfected with RSV and then challenged i.n. with OVA. AHR was measured on day 29. On day 30 the mice were sacrificed and BAL fluid, lungs and spleens were taken. All experiments were repeated at least twice.

### Determination of airway hyperreactivity (AHR)

AHR, expressed as enhanced pause (Penh), was measured in unrestrained mice by whole body plethysmography (Buxco, Troy, NY). Groups of mice (n = 4) were exposed for 5 min to nebulized PBS to establish a baseline then to increasing concentrations (6–50 mg/ml) of nebulized methacholine (MCh; Sigma, St. Louis, MO) in PBS. Challenges were done for 5 min followed by recordings of Penh for 5 min. The Penh values were averaged and expressed for each MCh concentration as a percentage of the PBS baseline reading.

### Bronchoalveolar lavage (BAL)

Lungs were lavaged using 500 μl of PBS and the BAL fluid was kept at 4°C until processed. BAL cells were centrifuged onto microscope slides by Cytospin (Shandon) and stained using the Hema3 kit (Fisher). Eosinophils, neutrophils and lymphocytes were counted from 5 different fields on 4 different slides from each group in a blinded fashion.

### Immunohistochemical analysis

Lung sections were stained with hematoxylin and eosin (H&E) and inflammation was assessed as disruption or denudation of the epithelial layer and infiltration of monocytes and lymphocytes into the perialveolar region. Lung sections were also stained with antibodies to the cell-adhesion molecule ICAM-1, the goblet cell marker Muc-5A, and the Th2 marker T1/ST2 (Santa Cruz BioTech, Santa Cruz, CA).

### Isolation of spleen cells and intracellular cytokine staining

Mice were sacrificed and single-cell suspensions were prepared from the spleens and cultured for 24 to 48 hrs in DMEM with 10% FBS. Splenocytes were incubated on anti-CD3-coated or OVA-coated plates for 24 h and then treated with Golgi-Stop for 4 hr to block the secretion of cytokines. Cells were surface-stained with FITC-anti-CD4 (Research Diagnostics, Flanders, NJ) and then subjected to intracellular staining with PE-anti-IFN-γ, -IL10 or -IL-4 antibodies (R&D Systems, Minneapolis, MN). Cells were analyzed by flow cytometry gated on CD4^+ ^cells. The percentages of CD4^+ ^cells secreting the respective cytokines are shown.

### Measurement of cytokine levels in BAL cells

Mice were sacrificed and lungs were lavaged with PBS. Aliquots of the BAL fluid were stored at -80°C until assayed. The concentration of IFN-γ in the BAL was measured by ELISA using a kit (R&D Systems, Minneapolis, MN), and the results were expressed as pg of IFN-γ per ml of BAL fluid.

### Apoptosis detection

Lung sections were processed for detection of apoptosis using the DeadEnd™ Fluorometric TUNEL System assay kit (Promega). Dewaxed sections were fixed and permeablized, then incubated with terminal deoxynucleotidyl transferase to label DNA ends with FITC-dUTP. Stained sections were photographed under a fluorescent microscope.

### Statistical analysis

All data were expressed as mean ± SEM. For comparison of two different groups, Student's *t *test was used. Differences between groups were considered significant at *P *< 0.05.

## Results

### FPS is more effective than FP or Sal alone in preventing AHR and lung inflammation

**Figure 1:**
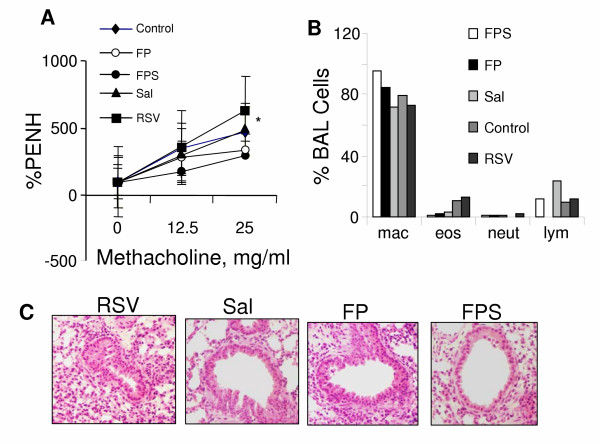
**FPS is more effective than FP or Sal alone in preventing AHR and lung inflammation**. Mice were sensitized with OVA and infected with RSV as described in *Methods*. From days 10 to 13 mice were treated daily with FP, Sal or FPS. AHR was measured on day 14 **(A)**. On day 15 the mice were sacrificed and a BAL cell differential was performed for macrophages (mac), eosinophils (eos), neutrophils (neut) and lymphocytes (lym) **(B) **and H & E stained lung sections were examined for histopathology **(C)**. Results shown are one representative experiment of two.

Mice were sensitized with OVA on day 1 i.p. and on day 7 and 9 i.n. On day 9 they were infected with 5 × 10^6 ^PFU RSV. From day 10 to 13 they were treated with 10 μg of FP, Sal or FP/Sal combination (FPS). AHR was measured as Penh on day 14 (Fig. 1A). On day 15 the mice were sacrificed, BAL was done and a differential cell count was performed on the BAL fluid (Fig. 1B). Lung sections were stained with H &E and examined for histopathology (Fig. 1C). Mice treated with FPS, FP and Sal all showed reduced eosinophil numbers but no change in neutrophils. Sal-treated mice showed an increase in the number of lymphocytes. Combination therapy resulted in a significant reduction in eosinophil number as well as lung inflammation compared to the other treatments. Lungs of allergen-sensitized and RSV-infected mice exhibited cellular infiltration with MNC and eosinophils (Fig. 1C).

### FPS is more effective than FP alone in reducing viral exacerbation of asthma

**Figure 2:**
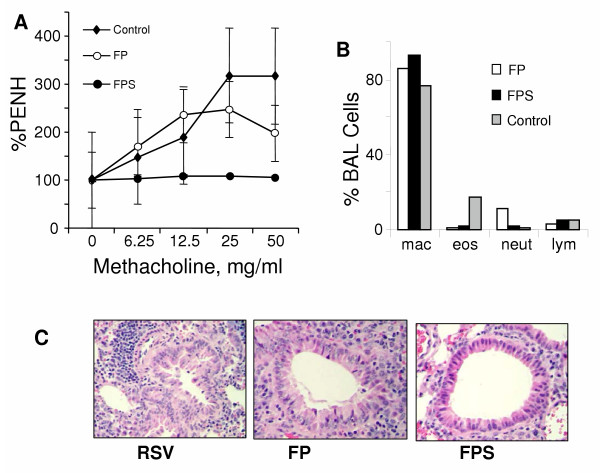
**FPS is more effective than FP or Sal alone in reducing viral exacerbation of asthma**. Mice were sensitized with OVA and infected with RSV as described in *Methods*. From days 21 to 27 mice were treated daily with FP, Sal or FPS. On day 28, they were reinfected with RSV and then challenged with OVA. AHR was measured on day 29 **(A). **On day 30 the mice were sacrificed and a BAL cell differential was performed for macrophages (mac), eosinophils (eos), neutrophils (neut) and lymphocytes (lym) **(B). **H & E stained lung sections were examined for histopathology **(C)**. Results are from one representative experiment of two performed.

Mice were sensitized with OVA i.p. on day 1 and on day 9, 12 and 14 i.n. On day 19 the mice were infected i.n with RSV (5 × 10^6 ^PFU). From day 21 to day 27, mice were treated i.n. with 10 μg of FP or FPS. On day 28 mice were reinfected with RSV and challenged with OVA. AHR was measured on day 29 and was lower in FPS-treated mice than those treated with FP or Sal alone (Fig. 2A). On day 30 the mice were sacrificed and BAL fluid was taken and analyzed for lymphocytes, eosinophils and neutrophils by differential staining. Treatment with FPS reduced the number of eosinophils and neutrophils compared to either drug alone (Fig. 2B). Lung sections showed extensive epithelial disruption and cellular infiltration in the case of RSV exposure without drug treatment. FP or S alone reduced the pathology somewhat but the combination FPS was significantly more effective (Fig. 2C). The results showed that FPS significantly attenuated AHR and inflammation, as seen by the decrease in eosinophils and lymphocytes and the reduced perialveolar damage compared to control group and mice treated with FP alone.

### Effect of FPS combination on cytokine and inflammatory marker expression

**Figure 3:**
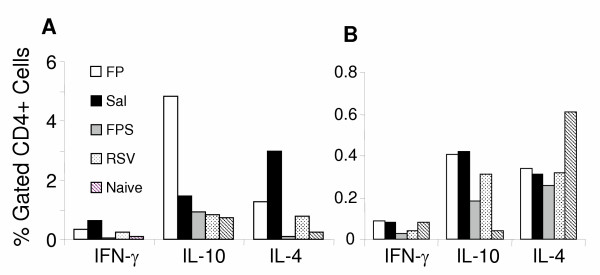
**FPS reduces production of cytokines by CD4^+ ^cells**. Mice were sensitized with OVA, infected with RSV then treated with FP, Sal or FPS as in Fig. 1A. They were sacrificed on day 15 and their splenocytes were examined for cytokine production by intracellular cytokine staining. Splenocytes were incubated on anti-CD3-coated plates (**A**) or with medium containing OVA (**B**) for 24 h and then treated with Golgi-Stop for 4 h to block cytokine secretion. Cells were surface-stained for CD4 and intracellularly for IFN-γ, IL10 or IL-4. The percent of CD4-gated cells producing the respective cytokine is shown. Results are from one representative experiment of two.

**Figure 4:**
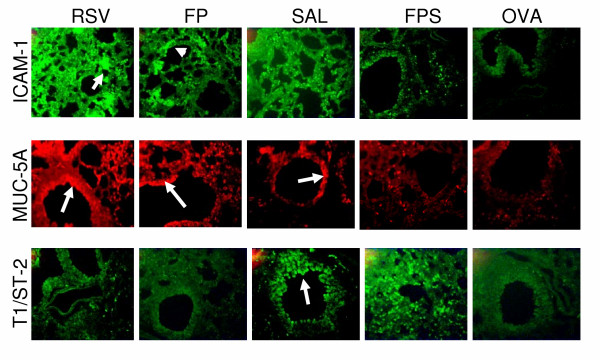
**FPS decreases expression of inflammatory markers in the lung**. Mice were sensitized with OVA, infected with RSV then treated with FP, Sal or FPS. They were sacrificed on day 15 and lung sections were stained for ICAM-1, Muc-5A or T1/ST2. Arrows indicate areas of staining by specific antibodies. Results are from one representative experiment of two.

To determine the effects of FPS therapy on T-cell cytokine secretion, mice were OVA-sensitized, infected with RSV and treated with FP, Sal or FPS. Mice were sacrificed after drug treatment and their splenocytes were examined for cytokine production by intracellular cytokine staining (Fig. 3A & B). Cells were stimulated with anti-CD3 in panel A and with OVA in panel B. FP increased the number of cells producing IL-10 and Sal increased cells producing IL-4, whereas FPS decreased the number of cells producing IL-10, IL-4 and IFN-γ. Lung epithelial cells respond to inflammatory cytokines by expressing specific cell markers such as the intracellular adhesion molecule, ICAM-1, and the mucin gene, MUC-5A. Fig. 4 shows the results of an immunohistochemical analysis for ICAM-1 and Muc-5A in mouse lung sections. RSV infection increased the expression of ICAM-1 and mucin, whereas treatment with FPS decreased their expression compared to control mice. Another correlate of lung inflammation is the infiltration of T lymphocytes into the perialveolar tissue. A Th2-specific marker, T1/ST2, was used to stain lung sections, and both Sal and FPS appear to stimulate infiltration of T cells of the Th2 type into the lung (**lower panel**, Fig. 4).

### FPS inhibits apoptosis of lung cells

**Figure 5:**
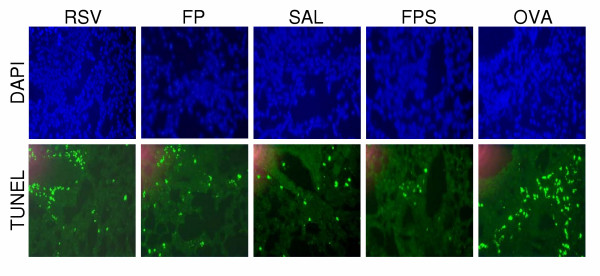
**FPS decreases apoptosis in the lung**. Mice were sensitized with OVA, infected with RSV then treated with FP, Sal or FPS. They were sacrificed on day 15 and lung sections were analyzed for apoptotic cells by TUNEL assay. DAPI stains nuclear DNA. Results are from one representative experiment of two.

Previous work from our lab documented the apoptosis of lung cells as a consequence of OVA-induced inflammation in OVA-sensitized mice. Here we sought to determine the effect of FP and Sal or the combination on this apoptosis. Mice were OVA-sensitized, RSV-infected and treated with the various drug combinations. At the end of treatment they were sacrificed, lungs were removed and sections were examined for apoptosis using the TUNEL (terminal deoxynucleotidyl transferase nick end-labeling) assay. Fig. 5 shows a representative experiment in which apoptosis was induced in a population of cells in the lungs of OVA-sensitized, RSV-infected mice. The extent of apoptosis was reduced by treatment with FP and Sal, and especially by FPS.

### FPS decreases RSV titer and increases IFN-γ production

**Figure 6:**
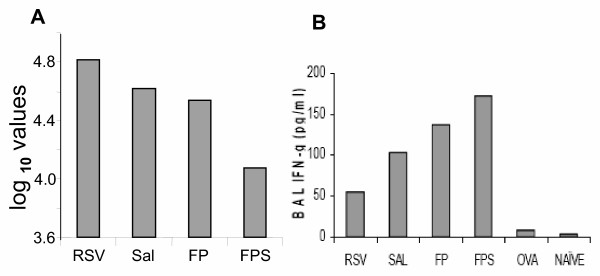
**FPS reduction in RSV titer correlates with increased IFN-γ production**. Two groups of mice were sensitized with OVA, and then prophylactically treated with FP, Sal or FPS prior to infection with RSV. Three days after infection, they were sacrificed. Homogenates of lungs from one group of mice were assayed for RSV titer, and BAL fluid from the second group was assayed for IFN-γ. Results are from one representative experiment of two.

Two groups of mice were sensitized with OVA i.p. and then prophylactically treated with FP, Sal or FPS prior to infection with RSV. Three days after infection, they were sacrificed. Lungs were removed from one group of mice, homogenized and the titer of RSV determined by plaque assay. BAL fluid was taken from the second group and analyzed by ELISA for IFN-γ. The decreased burden of virus in the lung tissue of FPS-treated mice was matched by an increase in secreted IFN-γ in BAL fluid (Fig. 6). Increased IFN-γ production could, at least in part, account for the reduction in RSV titer.

## Discussion

Our goal in this study was to determine if the combination of a corticosteroid and a long-acting β2-agonist was more effective in reducing RSV-induced asthma exacerbation than either drug alone. Here we demonstrate a reduction in lung inflammation in OVA-allergic mice infected with RSV after treatment with combined fluticasone and salmeterol. Eosinophil migration to the lung is characteristically seen during RSV-induced exacerbation of asthma [23], and both fluticasone and salmeterol reduced the eosinophil numbers in BAL fluid; but the combination of the two drugs was most effective. The basis of this synergy and whether the combination will be effective in infants and children affected by exposure to both viruses and allergens is unclear.

One important finding of this study is that irrespective of the frequency of RSV infection, the FPS combination therapy was effective and significantly decreased AHR as measured by % Penh. Also, in studies in which mice were infected twice with RSV, FPS decreased AHR more than FP alone. These results are in agreement with previous reports, where steroid or β2-agonists alone was found to be partially effective in RSV infection and viral induced asthma [24-26]. Consistent with the AHR data, there was a substantial reduction in pulmonary inflammation irrespective of the frequency of RSV infection. Of note is that in experiments where mice sensitized with OVA received one RSV infection, there was a decrease in eosinophils but an increase in lymphocytes. In contrast, OVA-sensitized mice that had two RSV infections before FP showed an increase in neutrophils. This is probably due to the resistance of neutrophils to corticosteroids and to the generation of a greater number of infiltrating neutrophils by the repeated RSV infection. The former possibility is consistent with the findings that neutrophilic asthma is resistant to treatment by FP [27]. This suggests that addition of the long-acting beta agonist is the agent primarily affecting recruitment of neutrophils in the combination.

Furthermore, single versus double RSV infection induced significant differences in the expression of specific cytokines such as IL-10 and IL-4. RSV infection in BALB/c mice has been shown to increase a variety of cytokines and chemokines including TNF-α, IFN-γ, IL-6, IL-4, IL-10, RANTES, macrophage inflammatory protein-1 alpha, and eotaxin [28]. Mice with a single infection showed an increased percentage of IL-10-producing cells perhaps indicative of greater stimulation of the innate immune system [29]. Treatment with salmeterol alone resulted in a significantly higher percentage of IL-4-producing cells characteristic of the Th2-type response observed in human infants suffering from acute RSV bronchiolitis [30] while FPS decreased both IL-10 and IL-4. The numbers of cytokine-producing cells in splenocyte cultures are in agreement with the increased number of Th2-like cells seen in lung sections from salmeterol-treated mice. In mice infected twice with RSV, FPS reduced IL-10 but not IL-4 compared to FP or salmeterol alone-. This is consistent with our previous finding that allergic mice with two RSV infections had an increase in the number of cells producing IL-4 and IL-13 [1]. Increased IL-10 production has been associated with recurrent wheezing in children hospitalized for RSV bronchiolitis [31,32].

Another significant observation is the differential effect of FP and Sal on inflammatory markers. FP treatment caused an increase in ICAM-1 expression whereas Sal showed a small decrease. Treatment with the combination resulted in a decrease of ICAM-1 expression. This observation is consistent with previous reports which showed that FPS combination decreased ICAM-1 expression in human lung fibroblast cells [33,34]. The expression of MUC-5A, which is a marker for mucus production, was also decreased by combined FPS treatment, but not significantly by either FP or Sal alone. Staining for the Th2-specific marker, T1/ST2 demonstrated the RSV-induced infiltration of Th2 cells. While treatment with FP increased ICAM-1 and MUC-5 expression, Th2 infiltration in lungs of FP-treated mice was decreased. In contrast, treatment with Sal did not inhibit Th2 infiltration. The combination FPS treatment decreased Th2 infiltration compared with Sal indicating that steroids and β_2_-agonists differentially affect inflammatory parameters, but that the two in combination can effectively reduce RSV-induced lung inflammation.

Apoptosis of lymphocytes in the lung is a characteristic feature of RSV bronchiolitis and may increase the severity of the disease [35]. Our results indicated that compared to lungs of mice sensitized with OVA in the presence or absence of RSV infection, lungs of mice treated with either FP or Sal and the combination FPS showed a decreased number of apoptotic cells. The FPS combination shows even fewer apoptotic cells compared to FP or Sal alone suggesting the greater effectiveness of the combination treatment. The reason for this is unclear, but it may be due to the fact that treatment with FP or Sal decreases infiltration of inflammatory cells to the lungs. This idea is consistent with the report that FPS increases apoptosis of peripheral blood T cells [36].

Treatment with FP plus Sal decreases lung inflammation and examination of lung homogenates for RSV revealed a reduction in virus titer in the mice treated with FPS, suggesting that the combination treatment exerts additional antiviral effects. The measurement of IFN-γ in BAL fluid showed an increased production of IFN-γ in the lungs of mice treated with FPS therapy. Thus, the observed reduction in RSV titer may be due to increased IFN-γ levels in the lungs induced by the FPS combination therapy.

Although a number of experimental approaches are under investigation for the treatment of RSV infection [37-40], the possibility that such infection can be managed using already available therapies is an important finding. The results of this -study show that OVA-sensitized mice receiving fluticasone plus salmeterol had a significant reduction in RSV-induced inflammation and RSV titers compared to those receiving the drugs individually. Our results suggest that the use of a combination therapy can be very effective in reducing virally-induced asthma exacerbations.

## Abbreviations

FP, fluticasone propionate; Sal, salmeterol; FPS, fluticasone plus salmeterol; RSV, respiratory syncytial virus; AHR, airway hyperreactivity; ICS, inhaled corticosteroids; OVA, ovalbumin; BAL, bronchoalveolar lavage; Th2, T helper cell type 2; URI, upper respiratory infection; PFU, plaque-forming units; i.p., intraperitoneal; i.n., intranasal; Penh, enhanced pause; MCh, methacholine.

## Competing interests

The author(s) declare that they have no competing interests.
